# Students’ perception of the learning environment and its relation to their study year and performance in Sudan

**DOI:** 10.5116/ijme.5af0.1fee

**Published:** 2018-05-24

**Authors:** Yasar Ahmed, Mohamed H. Taha, Salma Al-Neel, Abdelrahim M. Gaffar

**Affiliations:** 1 Medical Oncology Department, Sligo University Hospital, Ireland; 2 Medical Education Department, College of Medicine, Qassim University, Saudi Arabia; 3 Education Development and Research Centre, Faculty of Medicine, University of Gezira, Sudan; 4 Department of Medical Education, Nahda College, Sudan

**Keywords:** Students’ Perception, learning environment, study year, performance, Sudan

## Abstract

**Objectives:**

To evaluate students’ perceptions of the learning environment and to assess any differences in perception related to students’ performance and their year of study.

**Methods:**

A descriptive cross-sectional study was performed of 638 students from the second, sixth and tenth semesters at the Faculty of Medicine at Gezira University, Sudan. This study employed the Arabic-translated Dundee Ready Education Environment Measure. The main predictor variables were the study year and academic performance. Descriptive statistics and one-way analysis of variance with a post hoc Tukey-Kramer multiple comparisons test were used for data analysis.

**Results:**

The overall score for this study was 122/200 (SD=16.6), indicating a positive perception of the learning environment. The overall mean score was 109.94/200 (SD=21.2) for Semester 2 students, 122.9/200 (SD=20.29) for Semester 6 students, and 116.53 (SD=20.12) for Semester 10 students, reflecting a significant difference in students’ perceptions in different years of study (F _(2,2422)_ = 3.21, p=0.04). There was also a significant difference between the mean overall scores with respect to academic performance. High-achieving students’ mean DREEM score was 126 (SD=24.4); while low-achieving students’ mean DREEM score was 102 (SD=26.25) (F_(2,2453)_ = 3.53, p=0.029).

**Conclusions:**

High achievers’ perceptions of the learning environment are significantly better than those of low achievers. A significant difference was observed between students in different years of study. The differences in students’ academic performance should be further investigated, targeting specific domains. A large-scale study is required to differentiate between the weakness and the strength of each academic level.

## Introduction

The learning environment has been defined as everything that happens in the educational institute.^1^ It encompasses the educational, physical, social and psychological context in which students are immersed, and is thought to play a significant role in their professional and moral development.^2^ The concept of the learning environment has been gaining attention in medical education over the last three decades. This has been accompanied by rapid changes in the educational missions and directives of health professions around the globe, which include new programmes, curricula and strategies, and which have usually been undertaken to improve the whole learning environment for students.

The learning environment has a strong impact on students’ learning experiences and outcomes; it dictates what, how and why students learn.^3^ Also, it affects students’ level of enthusiasm and degree of learning effectiveness. The relationship between educational environment and students’ achievement has been a fertile area of investigation, and the literature provides a proven connection between educational environment and the valuable outcomes of students’ achievement, satisfaction and success.^4^ Furthermore, evidence from previous studies shows that students who perceive the educational climate favourably achieve higher academic success than those who perceive it negatively.^5^^,^^6^ The door was thus opened for conducting a considerable number of studies to examine students’ perceptions of their educational environment. In the field of health profession education, the literature supports the evidence of an association between students’ academic achievement and their perception of the learning environment. ^7^^-^^9^

To evaluate the learning environment in a health profession institute, it is crucial to use a wide-ranging, valid and reliable tool. That most widely used currently is almost certainly the Dundee Ready Education Environment Measure (DREEM),^10^ which was developed by an international Delphi panel in Dundee, Scotland. It is an international, validated tool that provides medical faculties with diagnostic help for measuring the overall state of affairs in the learning climate of their college, and it has been translated into various languages, including Arabic.^11^

DREEM^10^ is used as an evaluation measure to diagnose deficiencies in the current learning environment, to compare different groups’ experiences with the learning environment, and to compare the actual experiences of the educational environment with an ideal or expected one in the same group. It has also been used to examine the relationship between the learning environment and other measures.^12^ In a systemic review, Soemantri and colleagues concluded that DREEM is the best tool for evaluating medical students’ perceptions of the learning environment.^13^ It is also used as a diagnostic tool to assess learning environments, solve educational problems, and improve the efficacy of education. It can provide authorities with valuable information. Its main characteristics include its scientific content, practicality, sociality and optimality.^14^ It has been shown to be a reliable and valid tool for assessing students’ perception of their learning environment in diverse settings. DREEM has been used in many universities worldwide due to its optimal validity and reliability.

This study was conducted in the Republic of Sudan, at the Faculty of Medicine, University of Gezira (FMUG), which was established in 1975. It is an accredited community-oriented/community-based, problem-based school – the first such school in the Eastern Mediterranean region^15^ – and it seeks to ensure integration within the basic sciences and between the basic and clinical sciences. The faculty offers a five-year, ten-semester MBBS programme in three educational phases.^16^ It is now well recognised in Sudan and the region as an important yardstick in the development of medical education in the Eastern Mediterranean region.^17^

Assessment of the learning environment’s influence on student academic success and satisfaction with the current learning environment and experiences at FMUG is needed, as it is still a virgin area that requires exploration and investigation. However, there is a paucity of scientific papers from developing countries examining associations between the learning environment and academic achievement. The dearth of such assessment research makes it difficult to find out whether the learning environment in which students’ learning is taking place is satisfying to them or not.

As the educational environment strongly affects students’ achievements, satisfaction and success,^18^ it is important to gather feedback from students regarding their experience in the learning environment. Therefore, the objective of this study was to assess students’ perceptions of the educational environment at FMUG and to examine how this relates to their academic levels and achievements.

## Methods

### Study design and participants

This was a descriptive, cross-sectional study. The sample frame consisted of all undergraduate students studying in the academic year 2016–2017 at FMUG. Non-probability sampling was applied and all students in Semesters 2, 6 and 10 were invited to participate in the study to represent different academic levels.

Participants were registered, active students. Students who did not belong to the class of origin and those repeating were excluded. The response rate was approximately 75%; 638 out of 854 of the target population completed the inventory. The study was approved by the Ethics Committee of the Education Development Centre, FMUG, and conducted at the Faculty of Medicine, University of Gezira, Republic of Sudan.

### Study instrument and procedure

The study was conducted using the Arabic-translated Dundee Ready Education Environment Measure (DREEM). It contains 50 statements relating to a range of topics directly relevant to the educational environment, scored on a five-point Likert scale ranging from zero to four (4: strongly agree; 3: agree; 2: have no idea; 1: disagree; 0: strongly disagree). The 50 items have a maximum score of 200. However, 9 of the 50 items are negative statements and are reverse-scored.

The inventory encompasses five subscales: (1) students’ perception of learning (SPL) – 12 items; (2) students’ perception of teachers (SPT) – 11 items; (3) students’ academic self-perception (SASP) – 8 items; (4) students’ perception of atmosphere (SPA) – 12 items; and (5) students’ social self-perception (SSSP) – 7 items.

The criterion variables were the perceptions of the educational environment as measured by the overall and subscale scores of the DREEM inventory: SPL, SPT, SASP, SPA and SSSP.

The main predictor variables were the academic level and cumulative grade point average (CGPA). FMUG uses the credit hour system, where each course has a certain weight of credit hours. The CGPA was calculated by dividing the total points earned during the semesters attended at the university by the total number of credit hours of those semesters. Students with a CGPA of three and above were classed as high achievers, while those with CGPA of less than three were classified as low achievers. Academic achievement was based on the grade from the previous semester.

To ensure a high response rate, the Arabic version of the DREEM questionnaire was administered directly during lectures to all students studying in the second, sixth and tenth semesters at FMUG. Before directly administering the questionnaire, the background, importance and potential impacts of this study were explained to the students. They were informed in advance of the date of data collection for their group. Completion of the inventory was undertaken on a voluntary basis, and none of the information collected was identiﬁable, thereby maintaining data anonymity.

DREEM overall scores were interpreted using the guide developed by McAleer and Roff, which defines a score of 0–50 as ‘very poor’, 51–100 as indicating ‘plenty of problems’, 101–150 as being ‘more positive than negative’ and 151–200 as ‘excellent’.

### Data analysis

The DREEM items for the study sample were coded and analysed using the Statistical Package for the Social Sciences (SPSS) program, version 20. Descriptive statistics were applied to get the total mean and the means of the five subscales (SPL, SPT, SASP, SPA and SSSP). Results were expressed in the form of mean values of the total scale, subscales or items, and the maximum score percentages in each ordinal category were associated with a specific interpretation.^19^^,^^20^ One-way analysis of variance with a post hoc Tukey-Kramer multiple comparisons test was used to identify the significant differences between subgroups. Probability values of less than 0.05 were considered statistically signiﬁcant for all statistical tests.

## Results

The respondents were: 216 (33.8%) students of the second semester, 221 (34.6%) students of the sixth semester and 201 (31.5%) students of the tenth semester. Of the 638 respondents, 403 (63%) and 235 (37%) were low achievers and high achievers, respectively. Table 1 shows the characteristics of the respondents about academic level and performance.

**Table 1:** Characteristics of the respondents by academic level and performance (N=368)

Characteristic		N		(%)	
Semester of study/Academic level				
2^nd^ Semester	216		33.8	
6^th^ Semester	221		34.6	
10^th^ Semester	201		31.5	
Academic Performance according to CGPA				
< 2.5	158		24	
2.5-3.0	245		39	
Low Achiever	403		63.1	
3.1-3.5	140		21	
> 3.5	95		15	
High Achiever	235		36.8	

CGPA: Cumulative Grade Point Average

### Domain ratings and perception

The overall DREEM score for this study was 122/200. This score indicates that the learning environment in FMUG, overall, is perceived by students as more positive than negative (Table 2).

### Relationship between students’ perceptions and their academic year

There is a significant difference in students’ perception of their learning environment according to their study year. The overall mean DREEM scores were 109.94 (SD=21.2) for Semester 2 students, 122.91 (SD=20.29) for Semester 6 students and 116.53 (SD=20.12) for Semester 10 students. Table 3 shows the DREEM domains, with mean scores according to the year of study. The one-way analysis of variance (ANOVA) shows the significantly different perceptions of the learning environment among students in different study years (F_(2,2422)_ = 3.21, p=0.04).

When the total mean domain scores were compared between the groups, the mean scores for students’ perception of learning (SPL) (M=28.5, SD=6.51) and students’ perception of teaching (SPT) (M= 28.82, SD=5.03) were significantly higher for students in Semester 6 than for those in Semesters 2 and 10 (F_(2,2455)_ = 13.14, p <0.001). The mean scores for students’ academic self-perception (SASP) (M=24.99, SD=5.21) and students’ perception of atmosphere (SPA) (M=33.66, SD=6.21) were significantly higher for students in Semester 10 than for those in Semesters 2 and 6 (F_(__2,2455)_= 13.14, p <0.001). There was no statistically significant difference in students’ social self-perception (SSSP) among the three student groups (F_(__0.5222)_= 0.5222, p= 0.8111). These findings suggest that attitudes towards the learning environment differ according to the students’ year of study.

**Table 2:** The DREEM domains with total and individual scores, mean scores, and interpretations

Subscales	Mean	SD	Maximum score of Perception%	Interpretation by students' perception
SPL	24	5.79	50	Teaching is viewed negatively
SPT	30	4.9	68.1	Moving in the right direction
SASP	21	4.3	65.6	Feeling more on the positive side
SPA	33	3.22	68.7	A more positive atmosphere
SSSP	14	3.7	50	Not a nice place
Total	122	16.6	61	Overall perception is on the positive side

SPL=students’ perception of learning; SPT= students’ perceptions of teachers; SASP= students’ academic self-perceptions; SPA=students’ perception of atmosphere; SSSP= students’ social-self-perception; SD=standard deviation

### Relationship between perceptions and academic performance

Students with higher academic achievement had more positive perceptions regarding their education, while low-achieving students exhibited more negative perceptions of education. The Mann-Whitney test revealed significant differences in the mean DREEM scores according to academic performance. High-achieving students’ mean DREEM score was 126 (SD=24.4), while low-achieving students’ mean DREEM score was 102(SD=26.25) (F_(__2,2453)_ = 3.53, p=0.029).

Statistically significant differences were observed in three domains: students’ perception of teachers (SPT), students’ perception of atmosphere (SPA) and students’ social self-perception (SSSP) (F_(__2,2453)_ = 3.53, p=0.029). However, there was no noticeable difference in students’ perception of learning (SPL) or students’ perception of their academic performance (SASP). Table 4 shows the DREEM domains, with mean scores according to students’ academic achievement.

**Table 3:** DREEM domains, with mean scores by students’ year of study

Variables	2^nd^ Semester		6^th^ Semester		10^th^ Semester		p value
	Mean (%)	SD	Mean (%)	SD	Mean (%)	SD	
SPL	19.44 (40.5)	6.52	28.50 (59.3)	6.51	20.75 (43.2)	6.560	p< 0.05
SPT	20.33 (46.2)	5.20	28.82 (65.5)	5.03	20.64 (46.9)	4.937	p< 0.05
SASP	18.78 (55.5)	4.83	19.89 (62.1)	3.84	24.99 (78)	5.21	p< 0.05
SPA	25.28 (52.6)	6.94	27.75 (57.8)	5.99	33.66 (70.1)	6.21	p< 0.05
SSSP	15.11 (53.9)	3.83	17.95 (64.1)	3.70	16.49 (58.8)	3.52	ns

SPL=students’ perception of learning; SPT= students’ perceptions of teachers; SASP= students’ academic self-perceptions; SPA= students’ perception of atmosphere; SSSP= students’ social-self-perception; ns=Not Statistically Significant; SD= standard deviation

## Discussion

Although we had a good response rate (75%), nonresponsive bias might have affected the results. When the study was conducted, some students were busy with exam preparations, and others had returned home for the semester break.

### Effect of academic level/year of study on students’ perception

The students’ perception of their learning environment, in the study, varied according to their academic level. However, their perception did not follow a particular pattern as the students progressed in their studies. This finding is in agreement with several studies.^21^^-^^24^  The main factors affecting students’ perception of their learning environment include curriculum contents, teaching style, and handling of the education atmosphere.^21^ A study carried out in a problem-based learning (PBL) medical school found that students lost some of the neutrality they exhibited in the first year and became more critical of the learning environment as they progressed through the programme.^25^ The change to a full clinical environment may also contribute to students’ level of satisfaction.

Many of the items involved in the DREEM questionnaire are related to clinical encounters between doctor and patient, about which students in the second semester are supposed to be ‘naïve’. Two studies, from Saudi Arabia and Spain,^26^ suggest that students in their early courses in medical school do not have enough academic experience to contribute valid opinions about the educational process. Moreover, according to Till and colleagues, students on the initial health science courses are not sufficiently experienced to respond to the items related to clinical matters in the DREEM questionnaire, because the curricula followed during these first years include a large proportion of the basic sciences but offer little clinical training.^27^

The difference in perceptions between the second and tenth semesters could also be explained by the fact that, during the second semester, students still feel that they have fewer academic skills, while students on the higher courses are more mature and have developed better strategies for their learning.

The increase of the mean score for learning environment perception as students advance in their career differs from the findings of many authors, who found better perceptions of the learning environment during the early years of students’ career.^19^^,^^25^^,^^28^^-^^31^ These findings are in line with the results of studies carried out in Iran^32^ and Malaysia,^33^ which found a trend for reduced scores in the senior years. This could be explained by the enthusiasm and excitement of first-year students on successfully gaining entry into medical college.^29^ The literature indicates that the perception of students who have recently entered higher education is strongly influenced by the satisfaction of their expectations with respect to their career and by the difficulty or ease with which they adapt to their new university role.^21^^,^^34^ It can be assumed that the perception of an accelerating deterioration of the educational environment is due not exclusively to educational delivery but also to individual factors such as ageing, becoming more autonomous and becoming more critical.^35^^,^^36^ It is worth noting, however, that other scholars have postulated that the environment remains the same over time and that it is the students’ perceptions that change.^37^^-^^39^

**Table 4:** DREEM domains, with mean scores by the students' academic achievement

Item	High achiever		Low achiever		p-value
	Mean (%)	SD	Mean (%)	SD	
SPL	28.16 (58.6)	7.01	27.59 (75.4)	7.53	ns
SPT	29.13 (66.2)	5.85	26.40 (60)	6.30	p<0.001
SASP	21.04 (65.7)	4.87	20.74 (64.8)	5.83	ns
SPA	30.09 (61.3)	7.56	27.42 (57.1)	7.5	p<0.001
SSSP	18.53 (66.1)	4.29	17.50 (62.5)	4.9	p<0.05

SPL=students’ perception of learning; SPT= students’ perceptions of teachers; SASP= students’ academic self-perceptions; SPA= students’ perception of atmosphere; SSSP= students’ social-self-perception; ns=Not Statistically Significant; SD=standard deviation

### Effects of academic achievement on students’ perception

Research work was undertaken to use DREEM to identify various types of academic achiever and to predict the probable academic outcomes of particular individuals and subgroups in the absence of intervention.^11^ The results of the present study show a positive relationship between students’ perceptions of the learning environment and their academic performance. Students with higher academic achievements had more positive perceptions regarding their education. These findings are consistent with the results of similar studies that found that those with higher scores on their learning environment had higher CGPAs.^7^^-^^9^^,^^36^^,^^40^ Few studies showed a lack of relationship between students’ perceptions of the learning environment and their academic performance.^9^^,^^41^ A study conducted in Pakistan to correlate students’ CGPAs and their learning environment indicated that CGPA does not have an enormous impact on the mindset of students.^42^ This might be due to other intrinsic elements that could influence academic achievements, such as learning motivation, study habits and examination performance. According to the Bindu T study, study habits have a significant influence on academic achievement, with a significant difference between low and high achievers.^43^ There is a close relationship between poor study habits and underachievement. On the other hand, high achievers have been shown to have better study habits.^43^ Students’ perceptions of the learning environment influence their selection of learning approaches, which are correlated with their academic performance.^8^^,^^42^ This is supported by two studies, from Oman^44^ and Saudi Arabia,^45^ that indicate a positive correlation between learning environment, learning style and academic achievement.

One limitation of this study is that it involved medical students from a single institution, with a non-random sample, which constrains the generalisability of its findings to other medical schools. The study is also limited by the methodology and models used. Questionnaires cannot describe the full context; some factors affecting FMUG may have been omitted. There is a need to use qualitative methods, such as focus groups, observations or semi-structured interviews, to further explore the concept of educational environment. The study data were collected using a self-report questionnaire that offers a subjective assessment of the learning environment. Biased findings may have resulted due to the self-reported nature of the study.

## Conclusions

This study has illuminated some prominent findings regarding how undergraduate medical students in a Sudanese medical faculty perceive their educational environment. These findings suggest, overall, that the students’ perceptions of the educational environment at FMUG were on the positive side. Regarding the learning aspects, the students were most satisfied regarding academic atmosphere and academic self-perception, although they were very critical of the teaching and the social environment.

The students’ year of the study showed significant variations regarding the perception of the learning environment. A large-scale study taking each academic level separately is needed, to differentiate between the weaknesses and the strengths of each level.

In this study, there is a relationship between students’ perceptions of their learning environment and their academic performance as measured by CGPA. Students with higher academic achievements had more positive perceptions regarding their education. Academic achievement was significantly related to higher scores for perception of teaching, perception of atmosphere and social self-perception.

By looking at outcomes such as exam performance, it may be possible to quantify this impact and then harness the instrument as a curriculum development tool. The differences between the perceptions of high academic achievers and low achievers should be further investigated, targeting specific domains.

### Conflict of Interest

The authors declare that they have no conflict of interest.

## References

[1] McAleer S, Roff S. What is educational climate? Med Teach. 2001;23:333-334. 10.1080/01421590120063312 12098377

[2] Kennedy C, Lilley P, Kiss L, Littvay L, Harden RM. Curriculum trends in medical education in Europe in the 21st century. Association for Medical Education in Europe Conference. 2013 [cited 23 December 2017]; Available from: http://www.medine2.com/Public/docs/outputs/wp5/DV5.18.1_CURRICULUM_TRENDS_FINAL_REPORT.pdf.

[3] Bakhshialiabad H, Bakhshi M, Hassanshahi G (2015). Students' perceptions of the academic learning environment in seven medical sciences courses based on DREEM.. Adv Med Educ Pract.

[4] Lizzio A, Wilson K, Simons R (2002). University students' perceptions of the learning environment and academic outcomes: implications for theory and practice.. Journal Studies in Higher Education.

[5] Hamid B, Faroukh A, Mohammadhosein B (2013). Nursing students' perceptions of their Eeducational environment based on DREEM model in an Iranian University.. Malays J Med Sci.

[6] Genn JM (2001). AMEE Medical Education Guide No. 23 (Part 1): Curriculum, environment, climate, quality and change in medical education-a unifying perspective.. Med Teach.

[7] Mayya S, Roff S (2004). Students' perceptions of educational environment: a comparison of academic achievers and under-achievers at kasturba medical college, India.. Educ Health (Abingdon).

[8] Pimparyon S, Roff S, McAleer S, Pemba S. Educational environment, student approaches to e-learning and academic achievement in a Thai nursing school. Med Teach.2000;22:359-64.

[9] Al-Qahtani MF. Associations between approaches to study, the learning environment, and academic achievement. Journal of Taibah University Medical Sciences.2015;10:56-65.

[10] Al-Hazimi A, Zaini R, Al-Hyiani A, Hassan N, Gunaid A, Ponnamperuma G, Karunathilake I, Roff S, McAleer S, Davis M (2004). Educational environment in traditional and innovative medical schools: a study in four undergraduate medical schools.. Educ Health (Abingdon).

[11] Roff S. The Dundee ready educational environment measure (DREEM)--a generic instrument for measuring students' perceptions of undergraduate health professions curricula. Med Teach.2005;27:322-325. 10.1080/01421590500151054 16024414

[12] Miles S, Swift L, Leinster SJ (2012). The Dundee Ready Education Environment Measure (DREEM): a review of its adoption and use.. Med Teach.

[13] Soemantri D, Herrera C, Riquelme A. Measuring the educational environment in health professions studies: a systematic review. Med Teach.2010;32:947-952. 10.3109/01421591003686229 21090946

[14] Roff S, McAleer S, Ifere OS, Bhattacharya S (2001). A global diagnostic tool for measuring educational environment: comparing Nigeria and Nepal.. Med Teach.

[15] Hamad B (1985). Problem-based education in Gezira, Sudan.. Med Educ.

[16] Ahmed YA, Alneel S (2017). Analyzing the curriculum of the faculty of medicine, University of Gezira using Harden’s 10 questions framework.. J Adv Med Educ Prof.

[17] Osman WN, Algaili DE, Elsanousi M. Post Graduate studies in health professions education at Gezira-EDC: a pioneer step towards improving and reforming health professions education locally and regionally. Gezira Journal of Health Sciences. 2013;9:23-34.

[18] Lo C. How student satisfaction factors affect perceived learning. Journal of the Scholarship of Teaching and Learning.2010;10:47-54.

[19] Palmgren PJ, Lindquist I, Sundberg T, Nilsson GH, Laksov KB (2014). Exploring perceptions of the educational environment among undergraduate physiotherapy students.. Int J Med Educ.

[20] Swift L, Miles S, Leinster SJ. The analysis and reporting of the Dundee ready education environment measure (DREEM): some informed guidelines for evaluators. Creative Education. 2013;4:340-347.

[21] Cerón MC, Garbarini AI, Parro JF (2016). Comparison of the perception of the educational atmosphere by nursing students in a Chilean university.. Nurse Educ Today.

[22] Lokuhetty MD, Warnakulasuriya SP, Perera RI, De Silva HT, Wijesinghe HD. Students' perception of the educational environment in a medical faculty with an innovative curriculum in Sri Lanka. South-East Asian J Med Educ. 2010;4:9-16.

[23] Pimparyon P, Caleer SM, Pemba S, Roff S (2009). Educational environment, student approaches to learning and academic achievement in a Thai nursing school.. Med Teach.

[24] Makhdoom NM. Assessment of the quality of educational climate during undergraduate clinical teaching years in the college of medicine, Taibah University. Journal of Taibah University Medical Sciences. 2009;4:42-52.

[25] Nosair E, Mirghani Z, Mostafa RM (2015). Measuring students' perceptions of educational environment in the PBL program of Sharjah Medical College.. Journal of Medical Education and Curricular Development.

[26] Mojaddidi MA, Khoshhal KI, Habib F, Shalaby S, El-Bab ME, Al-Zalabani AH (2013). Reassessment of the undergraduate educational environment in College of Medicine, Taibah University, Almadinah Almunawwarah, Saudi Arabia.. Med Teach.

[27] Till H (2004). Identifying the perceived weaknesses of a new curriculum by means of the Dundee Ready Education Environment Measure (DREEM) Inventory.. Med Teach.

[28] Riquelme A, Oporto M, Oporto J, Méndez JI, Viviani P, Salech F, Chianale J, Moreno R, Sánchez I (2009). Measuring students’ perceptions of the educational climate of the new curriculum at the Pontificia Universidad Católica de Chile: performance of the Spanish translation of the Dundee Ready Education Environment Measure (DREEM).. Educ Health (Abingdon).

[29] Al-Ayed IH, Sheik SA (2008). Assessment of the educational environment at the College of Medicine of King Saud University, Riyadh.. East Mediterr Health J.

[30] Al-Naggar RA, Abdulghani M, Osman MT, Al-Kubaisy W, Daher AM, Nor Aripin KN, Assabri A, Al-Hidabi DA, Ibrahim MI, Al-Rofaai A, Ibrahim HS, Al-Talib H, Al-Khateeb A, Othman GQ, Abdulaziz QA, Chinna K, Bobryshev YV (2014). The Malaysia DREEM: perceptions of medical students about the learning environment in a medical school in Malaysia.. Adv Med Educ Pract.

[31] Al-Kabbaa AF, Ahmad HH, Saeed AA, Abdalla AM, Mustafa AA. Perception of the learning environment by students in a new medical school in Saudi Arabia: areas of concern. Journal of Taibah University Medical Sciences. 2012;7:69-75.

[32] Bakhshialiabad H, Bakhshi M, Hassanshahi G (2015). Students' perceptions of the academic learning environment in seven medical sciences courses based on DREEM.. Adv Med Educ Pract.

[33] Mohd Said N, Rogayah J, Hafizah A (2009). A study of learning environments in the Kulliyyah (faculty) of nursing, international islamic university malaysia.. Malays J Med Sci.

[34] Gale J, Ooms A, Newcombe P, Marks-Maran D (2015). Students' first year experience of a BSc (Hons) in nursing: a pilot study.. Nurse Educ Today.

[35] Rotthoff T, Ostapczuk MS, De Bruin J, Decking U, Schneider M, Ritz-Timme S (2011). Assessing the learning environment of a faculty: psychometric validation of the German version of the Dundee Ready Education Environment Measure with students and teachers.. Med Teach.

[36] Park KH, Park JH, Kim S, Rhee JA, Kim JH, Ahn YJ, Han JJ, Suh DJ (2015). [Students' perception of the educational environment of medical schools in Korea: findings from a nationwide survey].. Korean J Med Educ.

[37] Jiffry MT, McAleer S, Fernando S, Marasinghe RB (2005). Using the DREEM questionnaire to gather baseline information on an evolving medical school in Sri Lanka.. Med Teach.

[38] Edgren G, Haffling AC, Jakobsson U, McAleer S, Danielsen N (2010). Comparing the educational environment (as measured by DREEM) at two different stages of curriculum reform.. Med Teach.

[39] McKendree J (2009). Can we create an equivalent educational experience on a two campus medical school?. Med Teach.

[40] Kim H, Jeong H, Jeon P, Kim S, Park YB, Kang Y (2016). Perception study of traditional Korean medical students on the medical education using the Dundee ready educational environment measure.. Evid Based Complement Alternat Med.

[41] Ugusman A, Othman NA, Razak ZN, Soh MM, Faizul PN, Ibrahim SF (2015). Assessment of learning environment among the first year Malaysian medical students.. Journal of Taibah University Medical Sciences.

[42] Baig AU, Ahmed SH, Rizvi M, Ilyas MA, Ahmed M, Rehmani MS, et al. Comparison of educational environment perception of Dow Medical College students with CGPA. International Journal of Research. 2015;2:72-79.

[43] Bindu TV. Achievers and non-discrepant achievers in education. New Delhi: APH Publishing Corporation; 2007.

[44] Al-Zidgall L. Students' approaches to studying at the institute of health sciences. [Master of Medical Education Dissertation]. University of Dundee: UK; 1999.

[45] Al-Qahtani M. Approaches to study and learning environment in medical schools with special reference to the Gulf countries. [PhD Thesis]. Faculty of Medicine, Dentistry and Nursing: University of Dundee; 1999.

